# Training needs and influencing factors among rural-oriented general practitioners in Chongqing, China: a cross-sectional survey and latent profile analysis

**DOI:** 10.3389/fpubh.2026.1743744

**Published:** 2026-01-22

**Authors:** Xintao Huang, Xingxin Deng, Enze Liu, Chen Zhang, Min Tang, Xun Lei, Huisheng Deng

**Affiliations:** 1 Department of General Practice, The First Affiliated Hospital of Chongqing Medical University, Chongqing, China; 2 Department of Academic Affairs, College of General Practice (Fifth Clinical College) of Chongqing Medical University, Chongqing, China; 3 College of Public Health, Chongqing Medical University, Chongqing, China

**Keywords:** continuing medical education, continuous professional development, general practitioners, latent profile analysis, primary health care, training needs analysis

## Abstract

**Background:**

Rural-oriented General Practitioners (GPs) are a key workforce to alleviate physician shortages in central and western China and improve the quality of primary healthcare services. However, they currently face high attrition rates. Targeted and effective continuing medical education (CME) can partially improve their retention rates. Nevertheless, existing CME programs do not align with these GPs’ practical needs. This study aims to assess these GPs’ CME needs and associated influencing factors, providing evidence-based support for the development of CME curricula and related policy formulation.

**Methods:**

A cross-sectional survey was conducted via stratified sampling among 508 rural-oriented GPs across 38 counties of Chongqing from July to August 2025. Data were collected using the Chinese-validated version of the Hennessy-Hicks Training Needs Analysis Questionnaire, which had been translated and validated before implementation, and a researcher-designed general information questionnaire. The questionnaires covered basic demographics, perceived importance of professional skills, and self-rated proficiency scores. Descriptive statistics and comparative analysis were used to identify the overall priority training needs. Latent profile analysis was employed to identify the types of training needs, and multinomial logistic regression analysis was conducted to identify factors influencing training needs.

**Results:**

Rural-oriented GPs reported significant training needs across all 37 skills (*p* < 0.001). The most pronounced demand was observed in the *Clinical Research and Evidence-Based Practice* dimension (Mean difference 1.60, 95% CI: 1.49–1.72). Latent profile analysis identified three subgroups: low demand (46.46%), medium demand (41.14%), and high demand (12.40%). Logistic regression analysis showed that higher professional titles, higher monthly income, and engagement in research were associated with lower training needs, while having one child (compared to having no children) was associated with higher training needs.

**Conclusion:**

Training demands show heterogeneity across subgroups among rural-oriented GPs in Chongqing and are affected by multiple factors. A significant training gap exists with the most urgent need in *Clinical Research and Evidence-Based Practice*. A tiered, demand-driven CME system should be established, prioritizing the development of research course modules. This should be combined with mechanisms for professional title promotion and salary incentives, providing flexible learning options to enhance career prospects and retention. This study also provides a research framework that can be adapted in other regions and countries where such experience is scarce.

## 1 Background

The shortage of physicians in rural and remote areas is a persistent global public health challenge (1). While research indicates that inequalities in human resources for health (HRH) have gradually narrowed over the past three decades, they remain a major obstacle to achieving universal health coverage (UHC). Particularly in resource-limited rural regions, the lack of key personnel such as physicians results in significantly higher mortality from preventable diseases (2). Consequently, countries and regions around the world continue to actively explore and implement various policies to attract and retain qualified healthcare personnel and thereby ensure accessible and equitable primary healthcare services (3, 4). These policies typically comprise measures such as financial incentives, targeted training programs, career development support, and mandatory service schemes (5).

To address this challenge, China launched the Rural-oriented Medical Education Project in 2010 to tackle the critical shortage and maldistribution of primary care providers, particularly general practitioners (GPs). The program prepares undergraduate-level GPs to serve in primary healthcare institutions in central and western China, providing full tuition waivers and living allowances for a five-year curriculum. In return, participants must complete at least 6 years of service at designated primary healthcare institutions upon graduation (6, 7). Over the past decade, these rural-oriented GPs have completed their medical education and residency training, and are now engaged in practice across all areas of primary care, including clinical services, public health, medical education, and administration. By 2023, the Chinese government had trained over 80,000 rural-oriented GPs in more than 30 provinces (8). Research shows that they have played a notably positive role in addressing talent shortages and optimizing human resource structures at the grassroots level. As a new generation of physicians at primary healthcare institutions, these physicians have made important contributions to enhancing service accessibility and quality, improving doctor-patient relationships, and implementing national basic public health service programs (8–11).

However, as the first cohort of rural-oriented GPs gradually concludes their six-year compulsory service period, a critical challenge has emerged: retaining these professionals long-term. A cohort study of rural-oriented GPs showed that only 37.5% remained in primary care after fulfilling their service obligation (12). Even more alarmingly, a meta-analysis incorporating 49 nationwide studies indicates that the retention intention rate among rural-oriented GPs is as low as 16% (95% CI: 12–19%) (13). Existing research identifies multiple complex factors, such as inadequate compensation, heavy workloads, and limited professional recognition. Among these, limited career advancement opportunities are widely regarded as a core factor driving these highly trained professionals to leave (13–15). Rural-oriented GPs typically perceive poor career prospects at primary care institutions and limited access to cutting-edge medical knowledge and technologies. Over time, this can lead to professional burnout and identity crises (14, 15).

To address this challenge, evidence from international experience suggests that a well-designed and accessible continuing medical education (CME) and continuous professional development (CPD) system, closely integrated with physicians’ career development paths, can effectively enhance their professional competence and occupational identity. Such systems are a vital intervention for improving physician job satisfaction, enhancing job attractiveness, and ultimately increasing retention rates (16–18). In China, CME/CPD is also related to career advancement. The administrative authorities have clearly stipulated that CME qualification serves as an important basis for the appointment of professional technical positions and a key requirement for applying for higher-level qualifications. Meanwhile, CME/CPD can enhance the output of research and teaching achievements—another key criterion for career advancement—through sustained cultivation of these competencies (19). Therefore, participating in high-quality CME/CPD can help rural-oriented GPs accumulate promotion capital, improve job competency and satisfaction, and consequently reduce their intention to leave. However, China’s current CME/CPD programs for GPs largely mirror those designed for clinical specialists. This approach frequently results in a mismatch between training content and actual practical requirements, leading to low quality and inefficiency. Moreover, there are few CME/CPD courses and systems specifically designed for rural-oriented GPs (20). They may be a crucial modifiable factors contributing to the lack of essential skills, burnout and attrition (17), and understanding heterogeneous needs is the first step in realigning CME/CPD (21).

Therefore, to effectively tackle this challenge and enhance the long-term benefits of the policy, it is critical to establish a CME/CPD system that aligns with the career development needs of rural-oriented GPs. In response, this study represents a crucial first step in the systematic development of a CME/CPD program for rural-oriented GPs in Chongqing. Using quantitative data from a Training Needs Analysis (TNA) (22), we systematically evaluated the training needs of rural-oriented GPs. Unlike most prior studies that regarded GPs as a homogeneous group, this study identified subgroups with distinct training needs via Latent Profile Analysis (LPA) and investigated their key influencing factors. This provides an evidence-based foundation for developing targeted educational intervention strategies, maximizing the translation of knowledge into practice.

## 2 Methods

### 2.1 Study design

This study used a cross-sectional design and was conducted in Chongqing, China, from July to August 2025. The study population comprised rural-oriented GPs practicing in primary healthcare institutions in Chongqing. The research tools comprised the validated Chinese version of the Hennessy - Hicks Training Needs Analysis (HHTNA) Questionnaire, which was translated and validated by the research team prior to implementation as part of an educational intervention research PhD dissertation, and a demographic questionnaire developed by the research team.

### 2.2 Setting

Chongqing, a unique Chinese municipality that features “metropolitan areas, vast rural regions, extensive mountainous terrain, and large reservoir zones,” has distinct characteristics within China’s healthcare delivery system. Its pronounced urban–rural dual structure leads to differences among GPs in personnel composition, practice settings, and competency requirements. The differences in their CME/CPD needs reflect key challenges in the development of GP competencies across China’s urban–rural and regional disparities, providing valuable insights for many developing countries.

For this study, rural-oriented GPs refer to medical graduates trained under China’s Rural-Oriented Medical Student Training Program launched in 2010—also referred to as the Chinese Bonded Medical Program (8)—a national initiative designed to alleviate the shortage of physicians in central and western China. Globally, analogous strategies have been implemented, including the Physician Shortage Area Program (PSAP) in the United States and Thailand’s Collaborative Project to Increase Production of Rural Doctors (CPIRD) (23). Primary healthcare institutions were defined as those at the base of China’s three-tier healthcare system. These include urban community health service centers (and stations), rural township health centers, and village clinics. These institutions primarily deliver initial diagnosis of common and prevalent diseases among residents, the long-term management of chronic diseases, and the provision of basic public health services mandated by the state.

### 2.3 Sample size and recruitment

According to statistics from the School of General Practice at Chongqing Medical University (a designated rural-oriented GP training institution), as of 2025, 1,036 designated GPs had graduated since the first cohort in 2015 and remained employed in primary healthcare institutions across Chongqing Municipality. We calculated the minimum required sample size as 370 participants using a finite population-adjusted sample size formula. A total of 580 eligible GPs were contacted, 508 completed valid questionnaires, resulting in a response rate of 87.6% (508/580).

To gain a more comprehensive understanding of the training needs of rural-focused GPs across different regions, stratified sampling was employed. According to government documents, the 38 county-level districts in Chongqing were classified into three geographical regions—the central urban area, the Three Gorges Reservoir Area in northeastern Chongqing, and the Wuling Mountain Area in southeastern Chongqing—based on economic, demographic, and geographical conditions. Subsequently, eligible participants were sampled proportionally from each region to take part in the survey. In-person surveys were administered by the Alumni Association of the College of General Practice, accounted for 37.0% of the total respondents (*n* = 188). To improve accessibility given the large and geographically dispersed sample, electronic questionnaires were additionally distributed to some participants via the SoJump.com online survey platform for remote completion. These accounted for 63.0% of the total respondents (*n* = 320).

### 2.4 Questionnaire development

This study used the validated Chinese version of the HHTNA questionnaire (Ch-HHTNA). Both the original tool and its adapted version have demonstrated high reliability and validity in numerous prior studies and are acknowledged for use in training needs assessments within various healthcare systems globally (22). The original HHTNA questionnaire consists of 30 items organized into five domains: *research/audit, administrative/technical, communication/teamwork, management/supervisory,* and *clinical activities*. Respondents rate each item on two dimensions: its importance to their work (score A) and their current ability to perform it (score B). The training needs score is computed as the difference between these two scores. This quantitative metric identifies and prioritizes training gaps, effectively enabling training prioritization and resource allocation under limited resources (24). The tool is flexibly implemented using detailed manuals that guide on data collection, analysis, and item modification customized to research objectives. This ensures its suitability across diverse linguistic, cultural, and professional settings while maintaining strong psychometric properties (22, 25).

This study translated and adapted the tool with WHO authorization (25). First, a nine-member translation team was assembled in accordance with Beaton guidelines for translation and cross-cultural adaptation (26). The team consisted of two bilingual clinical doctoral students, two master’s students in GP, two medical education experts, two linguistics experts, and one methodology expert. The original HHTNA questionnaire underwent forward translation, back-translation, and synthesis. Subsequently, we adjusted questionnaire items per manual guidelines to align with the Chinese research context. Seven senior experts involved in Chinese GPs research (covering discipline development, medical education, health services, and clinical studies) were invited to provide written feedback. Expert consensus on cross-cultural modifications was reached via a modified Delphi method (27). Finally, a pilot test evaluated the clarity of questions, precision of item wording, the time needed to complete the questionnaire (9.37 ± 3.05 min), the display quality of the survey platform, and the effectiveness of navigation instructions. Ultimately, two items were removed and nine new items were added, fully conforming to the manual’s requirement: “a maximum of 8 items may be swapped for researcher-selected items of the researcher’s choice without invalidating the questionnaire and another 10 items may be added.” Additionally, eight items underwent minor revisions to increase their relevance to the daily practice of Chinese GPs, resulting in the Ch-HHTNA (see Supplementary file 1). Immediately following this, it was assessed for psychometric properties in 385 rural-oriented GPs by quota sampling in Chongqing (see Supplementary file 2).

The Ch-HHTNA is composed of 37 items, each of which respondents’ rate on two dimensions: the importance of the item to the respondent’s job performance (*Importance*: score A) and the respondent’s current proficiency in carrying out the item (*Proficiency:* score B). Respondents rated both dimensions on a 7-point Likert scale. Score A ranged from 1 = “Not at all important” to 7 = “Extremely important,” and score B ranged from 1 = “Not at all proficient” to 7 = “Extremely proficient.” In this study, for scale A, the Cronbach *α* coefficient was 0.977 in total and 0.826–0.958 for individual dimensions. For scale B, the Cronbach α coefficient was 0.974 in total and 0.780–0.957 for individual dimensions.

Demographic data were collected through a researcher-developed questionnaire, which included age, gender, educational background, professional title, career length and practice location, working content (e.g., clinical work, public health, research, teaching, administration; respondents could select multiple categories), marital and parental status, and monthly income level.

### 2.5 Quality control

Two research assistants independently extracted all data and performed double data entry to guarantee accuracy. Invalid questionnaires were excluded before data analysis. A questionnaire was deemed invalid if it met any of the following criteria: (1) incomplete responses; (2) aberrantly short completion time; (3) response pattern highly consistency (e.g., identical ratings across all items). To ensure methodological rigor and transparency in result reporting, this study adhered strictly to the STROBE Statement guidelines for cross-sectional research (see Supplementary file 3).

### 2.6 Statistical analyses

All statistical analyses were carried out using IBM SPSS Statistics version 29.0 and LPA was performed using Mplus version 8.3. Quantitative data were presented as mean ± standard deviation (M ± SD). Comparisons were made using t-tests and one-way ANOVA. For multiple comparisons, the Bonferroni correction-controlled Type I errors across multiple comparisons. Cohen’s d effect size was computed to evaluate the practical significance of differences between *Importance* (score A) and *Proficiency* (score B). The cutoff points for small, medium, and large Cohen’s d effect sizes were 0.20, 0.50, and 0.80, respectively. Categorical data were reported as frequencies (n) and percentages (%). Intergroup differences in unordered categorical variables were analyzed using chi-square tests, while ordered categorical variables were compared using the Kruskal-Wallis H test. The significance level was set at *α* = 0.05.

LPA used the mean training needs scores (score A–B) across five skill dimensions from the HHTNA questionnaire to identify subgroups of GPs with distinct training needs. Model fit was evaluated using the Akaike Information Criterion (AIC), Bayesian Information Criterion (BIC), and adjusted Bayesian Information Criterion (aBIC). Smaller AIC, BIC, and aBIC values signify better model fit. Lo–Mendell–Rubin Tests (LMRT) and Bootstrap Likelihood Ratio Tests (BLRT) were employed to compare the absolute fit of k-1 class models against k class models. A significant *p*-value supported k latent profiles, whereas a non-significant *p*-value supported k-1 latent profiles. Regarding model classification accuracy, entropy values > 0.80 were considered adequate (28). Optimal model selection is based on the alignment between statistical evidence and model interpretability. Potential categories were named according to the fluctuations seen in the mean line charts across each dimension.

Multinomial logistic regression analysis was used to further identify factors influencing the classification of rural-oriented GPs’ training needs. With the latent category grouping of rural-oriented GPs’ training needs as the dependent variable, univariate analysis was carried out to compare demographic variables across multiple groups. In accordance with the exploratory nature of this study and following the recommendations of Daniel (29) and Maier (30), the significance level for univariate analysis was relaxed to *α* = 0.10 to prevent the omission of variables with real associations. The selected independent variables then underwent multicollinearity analysis. Variables having Pearson correlation coefficients ≥ 0.7 were regarded as highly correlated, and those with variance inflation factors (VIF) > 10 were considered multicollinear. Variables with high correlation or multicollinearity were excluded (31). The remaining variables underwent logistic regression analysis (see Supplementary file 4 for the variable assignment table).

## 3 Results

### 3.1 Demographic characteristics

This study involved 508 rural-oriented GPs, with a mean age of 28.6 ± 3.4 years. Among them, 289 (56.89%) were female, 296 (58.27%) held intermediate or senior professional titles, 270 (53.15%) were unmarried, 275 (54.13%) worked in the central urban area, and 289 (56.89%) had monthly incomes of 3,000 ~ 6,999 CNY. The sample was highly educated, with 91.34% (*n* = 464) holding bachelor’s degrees, and most respondents (*n* = 438, 86.22%) were primarily engaged in clinical work. Additional demographic characteristics of the sample population are presented in detail in Table 1. To verify the data consistency between the two survey methods, we compared the in-person surveys group with the online surveys group in terms of key demographic characteristics and overall training needs scores. The results indicated no statistically significant differences in all aspects, suggesting that the data were free of survey method bias and enabled subsequent integrated analysis.

**Table 1:** Demographic characteristics of participants (*n* = 508).

Basic information	n	Ratio (%)
Gender		
Male	219	43.11
Female	289	56.89
Educational background		
Bachelor	464	91.34
Master	44	8.66
Professional title		
Junior or less	212	41.73
Intermediate or Senior	296	58.27
Career length (years)		
≤ 3	178	35.04
> 3 and ≤ 6	115	22.64
> 6 and ≤ 9	164	32.28
> 9	51	10.04
Marital status		
Unmarried	270	53.15
Married	238	46.85
Parental status		
No children	292	57.48
One child	172	33.86
Two or more children	44	8.66
Working content^#^		
Clinical work	438	86.22
Public health	91	17.91
Teaching	28	5.51
Administration	57	11.22
Research	20	3.94
Practice location		
The central urban area	275	54.13
The Wuling Mountain area	75	14.76
The Three Gorges Reservoir area	158	31.1
Monthly income level (CNY)		
< 3,000	43	8.46
3,000–6,999	289	56.89
≥ 7,000	176	34.65

^#^Participants could select more than one option.

### 3.2 Training needs of rural-oriented GPs

The results showed rural-oriented GPs rated all 37 skills highly for importance, with average scores ranging from 5.13 to 6.42. This indicates that rural-oriented GPs widely recognize their core responsibilities. The most critical skills identified were: *Diagnosing and treating common diseases, Mastery of basic clinical skills and interpreting ancillary test results,* and *Knowledge of basic pharmacology for rational drug use.* Paired t-tests indicated significant training needs for all skills (*p* < 0.001). Most skills had a Cohen’s d > 0.8 (see Supplementary file 5).

Table 2 presented the statistical results for the top 15 training needs that *Writing personal research papers* (mean difference 1.97, 95%CI: 1.82–2.13, Cohen’s d 1.08) had the highest training need, followed by *Designing research projects* (mean difference 1.89, 95%CI: 1.72–2.06, Cohen’s d 0.97) and *Accessing research resources* (mean difference 1.88, 95%CI: 1.72–2.04, Cohen’s d 1.00). By contrast, *Communicating with patients to promote joint decision-making* (mean difference 0.77, 95%CI: 0.67–0.87, Cohen’s d 0.69) and *Giving effective feedback to colleagues and physicians at higher-level hospitals* (mean difference 0.70, 95%CI: 0.61–0.80, Cohen’s d 0.63) had the lowest training needs.

**Table 2:** The top 15 training needs of rural-oriented general practitioners.

Rank	Dimensions	Skills	Training needs scores (A–B)		
			Mean difference (95% CI)	t-value	Cohen’s d
1	R	19. Writing personal research papers	1.97 (1.82–2.13)	24.36*	1.08
2	R	24. Designing research projects	1.89 (1.72–2.06)	21.84*	0.97
3	R	26. Accessing research resources (e.g., time, funding, information, equipment)	1.88 (1.72–2.04)	22.59*	1.00
4	R	14. Applying statistical methods to analyze personal research data	1.78 (1.62–1.93)	22.41*	0.99
5	R	23. Collecting and organizing relevant research information	1.69 (1.55–1.82)	23.94*	1.06
6	M	17. Managing time efficiently	1.64 (1.52–1.76)	27.00*	1.20
7	M	28. Understanding healthcare system changes, familiar with policy norms, and skilled at process optimization	1.64 (1.48–1.81)	20.00*	0.89
8	P	33. Implementing immunization programs while managing adverse reactions	1.63 (1.51–1.76)	25.30*	1.12
9	M	25. Collaborating effectively as a team with clear roles and responsibilities	1.63 (1.49–1.76)	23.63*	1.05
10	R	9. Identifying viable research topics based on clinical needs	1.63 (1.49–1.77)	22.51*	1.00
11	M	27. Participating in administration and management tasks	1.61 (1.48–1.75)	22.74*	1.01
12	P	31. Assisting in public health emergency responses	1.59 (1.46–1.72)	24.35*	1.08
13	M	21. Rationalizing the allocation of limited health resources	1.59 (1.46–1.73)	23.11*	1.03
14	R	15. Clinical teaching, demonstration, and evaluation of colleagues/students	1.59 (1.45–1.74)	21.73*	0.96
15	C	34. Managing common critical illnesses effectively	1.54 (1.40–1.67)	22.70*	1.01

CI, confidence interval. *p < 0.001. Dimensions column, C, Clinical Diagnosis aned Basic Skills; R, Clinical Research and Evidence-based Practice; M, Management and Organization; P, Public Health and Health Promotion; I, Communication and Interpersonal Relationships. The numbers in the skills column correspond to the entry numbers in Ch-HHTNA (see Supplementary file 1).

**Figure 1:**
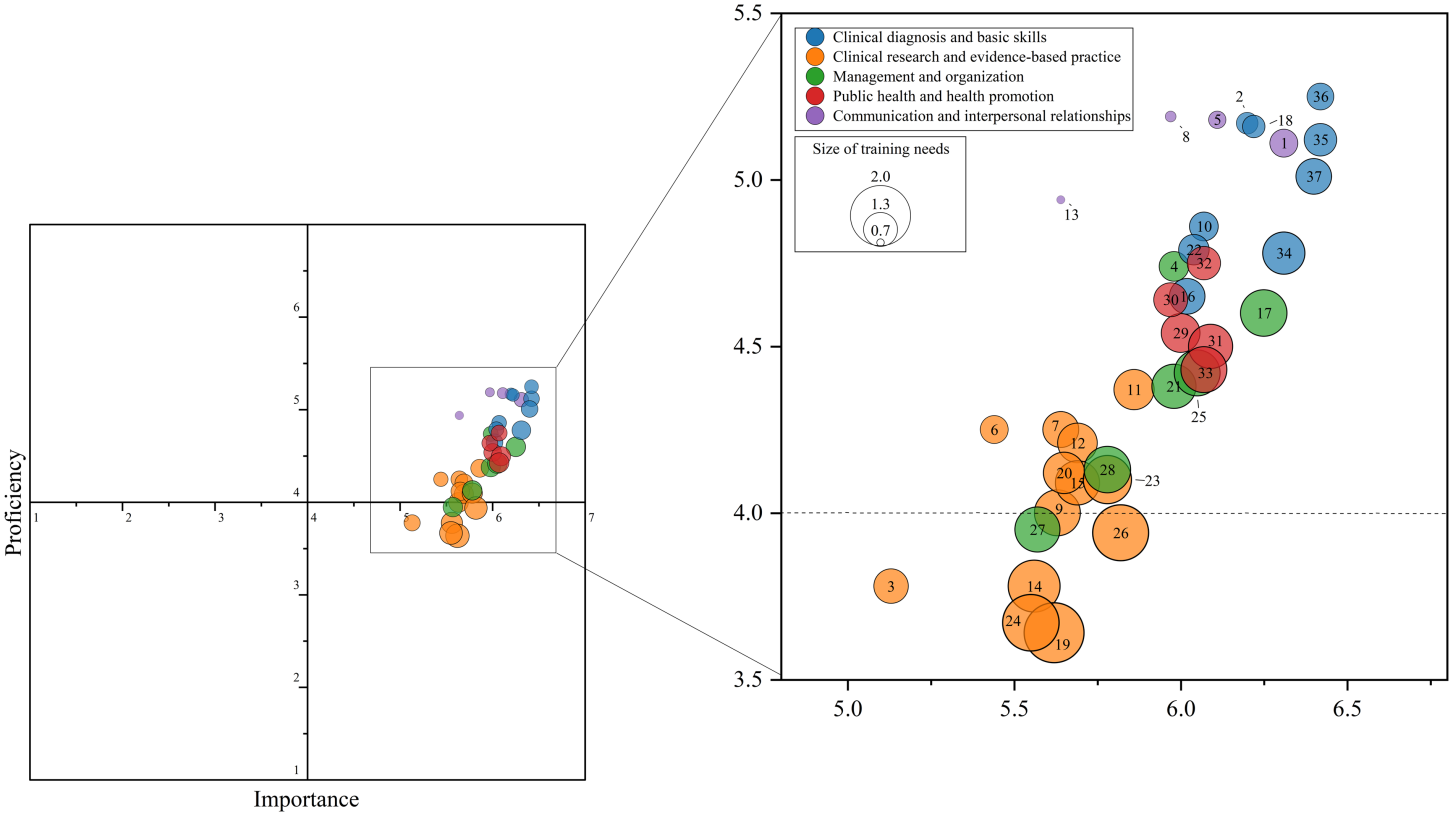
Four-quadrant bubble scatter plot of rural-oriented general practitioners training needs. The numbers in or next to the circles in the figure correspond to the entry numbers in Ch-HHTNA (see Supplementary file 1).

Figure 1 illustrates the distribution of importance and proficiency scores for the 37 skills using a four-quadrant bubble plot. Overall, most skills have importance scores concentrated between 5.0 and 6.5, while proficiency scores cluster between 3.5 and 5.0. This indicates that rural-oriented GPs generally consider these skills to be more important than their current proficiency, revealing a widespread training-need gap. Across the five dimensions, skills within the *Communication and Interpersonal Relationships,* and *Clinical Diagnosis and Basic Skills* dimensions are positioned in the first quadrant (high importance, relatively high proficiency). This shows a relatively solid foundation in these areas, although there is still room for improvement. Multiple skills in the *Clinical Research and Evidence-Based Practice* dimension fell in the fourth quadrant (high importance, low proficiency). This highlights the most urgent training needs, making this the top-priority area for training (the importance, proficiency, and training needs scores of 37 items and 5 dimensions in Supplementary file 5).

### 3.3 Latent profiles categories and characteristics of training needs

**Figure 2:**
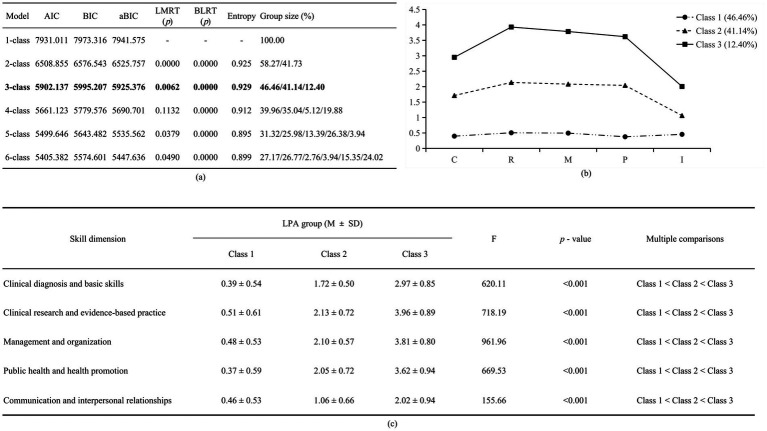
**(a)** Model fit evaluation for Latent Profile Analysis. Abbreviations: AIC, Akaike information criterion; BIC, Bayesian information criterion; aBIC, adjusted Bayesian information criterion; LMRT, Lo–Mendell–Rubin adjusted likelihood ratio test; BLRT, bootstrap likelihood ratio test. Note: bold figures highlight the selected class solution. **(b)** Parameters for the 3-class patterns. Note: The x-axis represents the 5 dimensions of training needs, the y-axis shows the estimated mean for each dimension. C, Clinical Diagnosis and Basic Skills; R, Clinical Research and Evidence-based Practice; M, Management and Organization; P, Public Health and Health Promotion; I, Communication and Interpersonal Relationships. **(c)** Average scores of training needs across various dimensions in latent classes. M ± SD, mean ± standard deviation. Class 1 = Low-Demand Group, Class 2 = Medium-Demand Group, Class 3 = High-Demand Group.

To explore the training needs of rural-oriented GPs, we tested latent profile models with one to six classes. Figure 2a presents the fit statistics for each latent profile model. As the model complexity increased, the AIC, BIC, and aBIC values decreased steadily. However, the rate of decline significantly slowed beginning from the 4-class model. Moreover, the LMRT *p*-value for the 4-class model was not significant, suggesting it was not better than the 3-class model. In terms of entropy values, all models had values exceeding 0.80, and the 3-class model achieved an entropy value of 0.929. Regarding group size, models with very small groups (e.g., <5%) generally exhibit lower stability and poorer interpretability. In this study, models with 4 or more categories all contained such small groups, while the 3-class model maintained a reasonable minimum group size (12.4%). Based on statistical fit and interpretability, the 3-class model was selected as the final solution.

Figure 2b shows that rural-oriented GPs’ overall training need profiles could be categorized into three latent classes. Class 1 (46.46%, *n* = 236) was labeled the Low-Demand Group, because it had the lowest and most consistent scores across all five skill dimensions, suggesting that this group has minimal training needs. Class 2 (41.14%, *n* = 209) had moderate training needs. It had relatively balanced scores across all skill dimensions except for *Communication and Interpersonal Relationships*, so we termed it the Medium-Demand Group. Class 3 (12.40%, *n* = 63) had the highest scores across all skill dimensions, especially in *Clinical Research and Evidence-Based Practice* and *Management and Organization*. This indicates that these physicians had the greatest training needs in these areas, thus it was named the High-Demand Group. Figure 2c presents statistically significant differences (*p* < 0.001) in training need scores across skill dimensions among the three subgroups. It shows that the High-Demand Group consistently scored higher than both the Medium-Demand Group and Low-Demand Group.

### 3.4 Univariate factors of training needs

Univariate analysis identified six variables associated with class membership (*p* < 0.10): Professional title, Career length, Parental status, Engaging in public health work, Engaging in research work, and Monthly income level (see Supplementary file 6). Further multicollinearity testing showed a significant correlation between Professional title and Career length (correlation coefficient 0.819, *p* < 0.01). The VIF values ranged from 1.022 to 3.550. Following discussion, Career length was excluded, and the remaining five variables were re-evaluated. The VIF values then ranged from 1.016 to 1.754. VIF values were <10 for all variables, indicating acceptable multicollinearity.

Previous studies have shown that urban–rural disparities in practice location affect training needs (32), leading to hypothesis testing after regional data integration. The results indicated that rural-oriented GPs in rural areas (the Wuling Mountain area and Three Gorges Reservoir area) had a slightly higher proportion in High-Demand Group (53.97%), while urban areas (the central urban area) had a slightly higher proportion in both Low- (56.78%) and Medium-Demand Group (53.59%). Nevertheless, none of these differences were statistically significant.

### 3.5 Multivariate factors of training needs

Using the latent profile classification of training needs for rural-oriented GPs as the dependent variable, we performed a multinomial logistic regression analysis with the indicators selected through univariate analysis as independent variables. Table 3 indicates that, rural-oriented GPs with intermediate/senior titles were significantly more likely to be in Medium-Demand Group compared to High-Demand Group (OR: 3.12, 95% CI: 1.16–8.39), and them more likely to be in Low-Demand Group compared to Medium-Demand Group (OR: 2.78, 95% CI: 1.15–6.88). Regarding monthly income levels, those with monthly incomes ≥ 7,000 CNY were significantly more likely to be in Low-Demand Group compared to High-Demand Group (OR: 1.52, 95% CI: 1.05–2.20) and Medium-Demand Group (OR: 3.18, 95% CI: 1.33–7.58). They were also more likely to be in Medium-Demand Group compared to High-Demand Group (OR: 4.83, 95% CI: 1.46–15.97). Moreover, rural-oriented GPs with monthly incomes of 3,000 ~ 6,999 CNY were significantly more likely to be in Low-Demand Group compared to Medium-Demand Group (OR: 3.31, 95% CI: 1.47–7.49). GPs engaging in research were significantly more likely to be in Low-Demand Group compared to Medium-Demand Group (OR = 5.47, 95% CI: 1.66–18.00).

**Table 3:** Multinomial Logistic regression analysis of factors influencing latent profile classification of rural-oriented general practitioner training needs.

Variable	Class 1 vs. Class 3				Class 2 vs. Class 3				Class 1 vs. Class 2			
	*β*	OR	95% CI	*p*-value	*β*	OR	95% CI	*p*-value	*β*	OR	95% CI	*p*-value
Professional title (ref: Junior or less)												
Intermediate or Senior	0.452	1.57	0.57–4.31	0.380	1.137	3.12	1.16–8.39	0.024*	1.022	2.78	1.15–6.88	0.039*
Parental status (ref: No children)												
One child	−1.334	0.26	0.11–0.63	0.003*	−0.704	0.49	0.25–0.96	0.016*	0.141	1.15	0.69–1.94	0.596
Two or more children	0.020	1.02	0.28–3.71	0.976	0.442	1.56	0.43–5.65	0.501	0.462	1.59	0.73–3.44	0.241
Engaging in public health work (ref: No)												
Yes	−0.797	0.45	0.18–1.14	0.091	−0.181	0.83	0.38–1.85	0.656	−0.333	0.72	0.18–2.96	0.645
Engaging in research work (ref: No)												
Yes	0.669	1.95	0.33–11.70	0.464	1.030	2.80	0.56–13.91	0.208	1.699	5.47	1.66–18.00	0.005*
Monthly income level (ref: <3,000 CNY)												
3,000–6,999 CNY	0.317	1.37	0.56–3.40	0.493	0.881	2.41	0.86–6.79	0.095	1.198	3.31	1.47–7.49	0.004*
≥ 7,000 CNY	0.418	1.52	1.05–2.20	0.045*	1.574	4.83	1.46–15.97	0.010*	1.156	3.18	1.33–7.58	0.009*

OR, odds ratio; CI, confidence interval. *p < 0.05. Class 1 = Low-Demand Group, Class 2 = Medium-Demand Group, Class 3 = High-Demand Group.

In terms of parental status, GPs raising one child were significantly more likely to remain in High-Demand Group compared to Medium-Demand Group (OR: 0.49, 95% CI: 0.25–0.96) and Low-Demand Group (OR: 0.26, 95% CI: 0.11–0.63), whereas having two or more children did not demonstrate a significant association with increased training demands. Since prior research shows that family responsibilities exacerbate work–family conflict and increase mental health risks, particularly among female physicians (33). This may further impede their access to training resources. This may further amplify their perception of disparities in the importance and proficiency of skills, thereby making training needs more pronounced. This hypothesis hint gender might change the strength or direction of the association between the ‘one child’ variable and training needs, we performed a gender interaction test on this variable. The comparison of goodness-of-fit between the interaction model and the main effect model reveals that incorporating the gender interaction term did not improve the model’s explanatory power. Moreover, in the interaction model, the gender interaction term had no statistically significant effect in either the Low- vs. High-Demand Group (OR: 1.21, 95% CI: 0.45–3.28) or the Medium- vs. High-Demand Group (OR: 0.89, 95% CI: 0.36–2.19).

## 4 Discussion

This is among the first cross-sectional studies from China to investigate the CME/CPD training needs of rural-oriented GPs, identifying substantial professional development needs, subgroup heterogeneity, and associated predictors. The findings not only offer new perspectives on the professional challenges faced by this distinctive group but also provide crucial data support for establishing a targeted CME/CPD system.

### 4.1 Characteristics and practical significance of rural-oriented GPs’ training needs

First, we found that rural-oriented GPs in Chongqing reported extensive training needs, based on their self-assessments of skill importance and proficiency. The importance scores for all 37 skills were significantly higher than their proficiency scores. This indicates a pervasive self-perceived gap between the importance GPs assigned to their responsibilities and their actual performance. It also emphasizes the urgency and necessity of implementing systematic CME/CPD programs. Among the skills, the *Communication and Interpersonal Relationships*, and *Clinical Diagnosis and Basic Skills* showed relatively higher proficiency, consistent with Wang (34) 2022 survey of GPs in Shanghai, China. They found that these competencies met routine community demands of community practice, while teaching skills were the primary area of deficiency. The high proficiency in patient management and communication may be related to their status as core GP functions (34). However, prior research has noted that self-assessment alone is limited for evaluating CME/CPD needs. Combining these with objective tools (e.g., performance-based tests, peer review) would provide more comprehensive and accurate understanding of GPs’ competencies and training requirements (35, 36). Therefore, future research should incorporate multidimensional evaluation methods to assess the proficiency of relevant skills among GPs.

The most pressing training needs were in the *Clinical Research and Evidence-based Practice* dimension, especially skills such as *Writing personal research papers, Designing research projects,* and *Accessing research resources*. This may reflect that these well-trained professionals, educated through systematic higher medical education and standardized residency training. As medical knowledge evolves rapidly, modern medical education increasingly emphasizes critical thinking and evidence-based practice in clinical work to optimize patient care and the quality of medical decision-making (37). Similarly, Liu’s (32) national survey of primary care providers found that 77.8% wanted to improve their teaching and research capabilities. However, China currently has insufficient CME/CPD courses in these areas. A retrospective analysis by You (38) of China’s national CME programs for GPs revealed overemphasis on Patient Care/Medical Knowledge while neglecting core competencies such as Practice-Based Learning and Improvement. This restriction, coupled with the importance of research output for physician promotion in China (39), creates strong incentives for research-related training.

### 4.2 The group heterogeneity of rural-oriented GPs’ training needs and its influencing mechanisms

Through LPA, we identified three distinct subgroups with significant differences among rural-oriented GPs in Chongqing: Low-, Medium- and High-Demand Groups. This finding challenges the traditional view of GPs as a homogeneous group, revealing the inherent heterogeneity in their training needs. Simultaneously, this study diverges from previous findings among all GPs (32). Specifically, although in High-Demand Group, the number of rural-oriented GPs working in rural areas slightly exceeds that of their urban counterparts, statistical tests showed no significant difference. This phenomenon may be ascribed to the fact that these GPs received standardized and high-quality medical education and residency training before returning to primary healthcare institutions. This finding further validates the effectiveness of rural-oriented medical education policies in narrowing regional disparities in the core competencies of GPs and addressing inequalities in HRH. This is also inseparable from the Chinese government’s efforts in recent years to narrow the urban–rural healthcare gap (40).

Further analysis explored multidimensional factors influencing the classification of training demand. Regression analysis showed that professional title, monthly income, research engagement, and single-child parental status were key variables differentiating these demand groups. Specifically, higher professional titles, higher monthly income, and participation in research were negatively associated with higher training needs—a finding that defies conventional wisdom. This phenomenon may stem from the fact that GPs with higher titles—and thus more clinical experience—had greater proficiency and thus lower training requirements. Wang (34) research also demonstrates this tendency. This suggests that training resource allocation for rural-oriented GPs should be tailored according to their professional title and clinical experience. Additionally, primary healthcare institutions with research support capabilities typically offer more abundant CME/CPD resources, professional development opportunities, and institutional support. GPs working in such settings are more likely to have filled critical skill gaps through on-the-job learning or targeted training, thus reducing their training requirements. Moreover, participation in research itself may directly enhance GPs’ professional competence and self-efficacy, potentially narrowing the gap between the perceived importance and proficiency of skills, which in turn lessens their training demands. This aligns with Liu’s (32) findings on hierarchical disparities in training access in the distribution of training opportunities. This underscores the need for equitable regional and institutional resource allocation.

We also discovered that having one child was positively correlated with higher training needs, whereas having two or more children did not demonstrate a significant association with increased training demands. This finding may be explained: First, the parenting duties of single-child parents may divert GPs’ time and energy from professional development, resulting in greater disparities in the importance and proficiency of skills. In contrast, GPs with two or more children may benefit from more flexible care arrangements due to their experience in caring for the first child, thus alleviating such conflicts. However, as the sample size of GPs with two or more children is relatively small (*n* = 44, 8.66%), future studies with larger sample sizes are required to validate this hypothesis. Meanwhile, based on previous research, we proposed the hypothesis that traditional gender roles lead to more pronounced effects of fertility status on the training needs of female physicians. In this study, we investigated whether this association varies by gender but did not observe a significant interaction effect between gender and single-child parental status on training needs. This suggests that the impact of having one child on training needs is consistent across genders among rural-oriented GPs in Chongqing, China. This might be associated with the gender-equal family division of labor promoted by the country and region in recent years, or it could be attributed to the small sample size of the subgroup analysis. In summary, training programs should account for family circumstances and offer flexible scheduling to reduce participation barriers.

This study identified training needs from a competency-gap perspective for specific skills based on GPs’ self-assessments of the importance and proficiency of their core competencies. The inferences drawn can guide the provision of more rational and effective external training resources. Yet intrinsic factors—motivation, burnout, and career aspirations—also influence training outcomes (41, 42). The CME/CPD curriculum and system that matches the needs may advance the ultimate impact of CME/CPD on retention by influencing a series of intermediate variables (e.g., perceived workload, job burnout, and occupational identity). By reducing the perceived workload through enhanced capabilities and improved work efficiency, and job burnout is alleviated as personal accomplishment increases through the acquisition of new knowledge and skills. This implies that future development of the training system must consider both filling competency gaps and stimulating intrinsic motivation.

### 4.3 Policy and practical suggestions

This study indicates that future CME/CPD program design should shift from a one-size-fits-all approach to a more targeted, personalized, and tiered curriculum system (38). The traditional one-size-fits-all CME/CPD model assumes uniform training requirements for all GPs, resulting in standardized content that overlooks individual or subgroup differences. It squanders limited educational resources by compelling GPs with low training needs to undergo redundant training, while failing to meet the urgent requirements of those with high training demands. In contrast, a targeted, personalized, and tiered curriculum system—by incorporating subgroup-specific characteristics into framework design, curriculum development, and implementation methods—can optimize resource allocation, improve training relevance, and promote knowledge translation. This approach maximizes support for the professional development of rural-oriented GPs and may enhance staff retention rates by reducing perceived workload, job burnout, etc. Accordingly, we propose the following policy recommendations:

First, establish an integrated, subgroup-specific CME/CPD framework for rural-oriented GPs. Health administrative departments should partner with medical universities to develop a progressive, integrated CME/CPD pathway that covers the entire career of rural-oriented GPs (8). This pathway should integrate subgroup characteristics and specify learning objectives, content, and delivery methods for each career stage. For example, for the High-Demand Group (mostly junior titles, lower income, and one child), prioritize gap-filling intensive training, particularly in clinical research and evidence-based practice (e.g., simplified research methodology, grassroots disease research design, and academic paper writing). For the Low-Demand Group (predominantly senior titles, higher income, and research engagement), the framework should focus on competence refinement—e.g., advanced clinical decision-making for complex cases or cutting-edge knowledge updates—avoiding redundant foundational training. Integrating this framework with promotion, compensation, and performance systems to create a predictable and attainable career development blueprint that addresses the unique motivations and barriers of each subgroup.

Second, course modules should integrate demand-driven and competency-based approaches (38). For example, to address the shortcomings in clinical research and evidence-based practice capabilities, focus on simplified research methodologies, grassroots-relevant disease research, and online research collaboration. Complementary support mechanisms—such as career development counseling, burnout interventions, and personalized growth planning (43, 44), could mitigate burnout and its negative effects on training engagement. This would strengthen professional identity, boost career expectations, and stimulate intrinsic motivation to address skill gaps.

Third, leveraging technology to develop innovative models and establish accessible online learning platforms (45). This can help overcome geographical barriers and ensure equitable access to high quality resources. At the same time, offer flexible formats (e.g., online courses, micro-learning) and scheduling options (32, 38), would reduce barriers to training participation, particularly for physicians with childcare responsibilities.

Moreover, China currently lacks both a focus on rural-oriented GPs’ CME/CPD and a comprehensive system for all GPs. International experience shows that mature systems in developed countries integrate curriculum design, quality control, management systems, and physician recertification processes (46). Future research should therefore explore CME/CPD methods tailored to China’s context (47).

### 4.4 Implications and limitations

A key strength of this study is its focus on rural-oriented GPs—a crucial group in China’s primary healthcare strategy. It is among the first to systematically explore these CME/CPD needs and their predictors. We propose strategies to advance GP education within China’s primary healthcare system via a “research-driven education that feeds back into practice” model. Our approach offers insights for other regions and countries with similar healthcare systems. Furthermore, research suggests health workforce databases that align training with population health priorities and skill gaps can support progress toward UHC (48). Our tools and methods could inform the development of such a database.

This study has several limitations. First, the self-reported data may be prone to recall bias, social desirability bias, and Dunning-Kruger effect (49). Future research should incorporate perspectives from other stakeholders (e.g., healthcare administrators, nurses, patients) and objective measures (e.g., standardized tests, peer review, patient feedback, clinical simulations). This would integrate health system planning with population health needs, yielding more comprehensive and reliable conclusions (22, 36). Second, as a quantitative study, it focused on quantifying relationships and patterns among variables. It may not fully capture respondents’ motivations, implicit needs, or subjective perceptions. Qualitative research should explore these perceptions and needs in greater depth. Integrating qualitative and quantitative findings would provide more comprehensive, context-sensitive insights. Third, Given marked regional disparities in disease spectra, such variations directly translate into pronounced heterogeneity in clinical service priorities, competency gaps, and training needs among rural-oriented GPs across different regions (38). Consequently, the study findings and recommendations from a single region cannot be fully applied to similar populations in other parts of China and other developing countries. As mentioned earlier, this study proposes a replicable research paradigm that can be adopted by various regions to conduct targeted assessments of rural-oriented GPs’ training needs and formulate localized, tailored training strategies. Future research could expand the sample scope to clarify the training needs of rural-oriented GPs in diverse contexts, identify cross-disciplinary and cross-regional commonalities and specificities in such needs, and ultimately promote the development of more robust regional and national training strategies and policies.

This study represents a first step toward developing a CME/CPD program for rural-oriented GPs in Chongqing, China. Future research will focus on establishing a competency-based CME/CPD system for this population. Additionally, although CME/CPD is theoretically and perceptually one of the core strategies to tackle the shortage of medical staff, the scientific evidence base regarding its mechanisms, impact pathways, and practical effects remains is still limited (50). Future research urgently needs more rigorous designs to fill current knowledge gaps. For example, longitudinal studies can be conducted to track training outcomes and explore long-term mechanisms, and validated measurement tools can be employed to track dynamic changes in perceived workload, burnout levels, and other key metrics after CME/CPD interventions, thereby providing more reliable decision-making support for policymakers and program managers. These efforts aim to help achieve the goals of “retaining, effectively utilizing, and rapidly developing” rural-oriented GPs. Such a system would offer strong academic support and practical models for the sustainable development of China’s primary healthcare system.

## 5 Conclusion

This study demonstrates that the training needs of rural-oriented GPs in Chongqing are not uniform but fall into three distinct, predictable profiles—namely, the Low-, Medium- and High-Demand Groups—which are significantly influenced by professional title, income, research activity, and single-child parental status. Furthermore, this TNA confirmed the existence of widespread competency gaps among this cohort. The *Clinical Research and Evidence-Based Practice* skills showed the greatest deficiency and should be prioritized in training. Therefore, we recommend that health policymakers and educational institutions establish a tiered, needs-based CME/CPD system integrated with promotion and incentive mechanisms. Priority should be given to developing needs-based curriculum modules aligned with job competencies, supplemented by career support mechanisms. Online platforms and flexible learning formats can reduce participation barriers.

Future research should incorporate objective competency assessments, qualitative methods, and longitudinal follow-up to evaluate training effectiveness and its impact on retention. These efforts are expected to address the career challenges facing rural-oriented GPs, strengthen their professional competencies and long-term retention intentions, and ultimately sustain the success of rural-oriented medical education policies while supporting the sustainable development of China’s primary care workforce.

## Data Availability

The raw data supporting the conclusions of this article will be made available by the authors, without undue reservation.
