# Supplementary material for: Training needs and influencing factors among rural-oriented general practitioners in Chongqing, China: a cross-sectional survey and latent profile analysis
Source: Front Public Health. 2026 Jan 22;14:1743744. doi: 10.3389/fpubh.2026.1743744 (PMC12872738; doi:10.3389/fpubh.2026.1743744)
Supplement: Supplementary file 1 [file Table_1.DOCX]

| **Supplementary File 1** The Chinese Hennessy-Hicks Training Needs Analysis (Ch-HHTNA) questionnaire | | |
| --- | --- | --- |
| In order to do your job effectively, you need to have the relevant skills. Listed below is a list of skills that you may use in your job. Please look at each of these skills and rate them by writing the appropriate number in the box. The first rating (A) is about how important the skill is to your success in completing your job; the second rating (B) is about how proficient you currently are in using the skill. | | |
| A: How important is this skill to your success in completing your job? (i.e., importance)  Rating 1-7: Not at all important = 1, very important = 7 | | |
| B: How proficient do you think you are currently using this skill? (i.e., proficiency)  Rating 1-7: Not proficient = 1, Very proficient = 7 | | |
| Skills | A (Importance) | B (Proficiency) |
| 1.Establishing a good relationship with patients |  |  |
| 2.Utilizing information technology for electronic documentation and clinical data processing |  |  |
| 3.Critically evaluating published research |  |  |
| 4.Assessing the effectiveness of one's own work |  |  |
| 5.Collaborating harmoniously with colleagues and doctors in higher-level hospitals |  |  |
| 6.Explaining personal research findings |  |  |
| 7.Applying others' or your own research findings to clinical practice |  |  |
| 8.Communicating with patients to promote joint decision-making |  |  |
| 9.Identifying viable research topics based on clinical needs |  |  |
| 10.Providing specialized treatment and developing health management plans for patients |  |  |
| 11.Incorporating innovative ideas and cutting-edge knowledge into clinical practice |  |  |
| 12.Conducting literature searches based on evidence-based medicine |  |  |
| 13.Giving effective feedback to colleagues and physicians at higher-level hospitals |  |  |
| 14.Applying statistical methods to analyze personal research data |  |  |
| 15.Clinical teaching, demonstration, and evaluation of colleagues/students |  |  |
| 16.Assessing patients' psychological and social needs |  |  |
| 17.Managing time efficiently |  |  |
| 18.Operating medical equipment (e.g., electronic sphygmomanometer, spirometer) using computerized office software |  |  |
| 19.Writing personal research papers |  |  |
| 20.Participating in health promotion-related research implementation |  |  |
| 21.Rationalizing the allocation of limited health resources |  |  |
| 22.Assessing patient’s clinical needs |  |  |
| 23.Collecting and organizing relevant research information |  |  |
| 24.Designing research projects |  |  |
| 25.Collaborating effectively as a team with clear roles and responsibilities |  |  |
| 26.Accessing research resources (e.g., time, funding, information, equipment) |  |  |
| 27.Participating in administration and management tasks |  |  |
| 28.Understanding healthcare system changes, familiar with policy norms, and skilled at process optimization |  |  |
| 29.Managing health records for key community populations (elderly, children, pregnant women, chronic disease patients) |  |  |
| 30.Providing health education to patients and the public using scientific knowledge |  |  |
| 31.Assisting in public health emergency responses |  |  |
| 32.Recognizing, reporting, and managing infectious diseases |  |  |
| 33.Implementing immunization programs while managing adverse reactions |  |  |
| 34.Managing common critical illnesses effectively |  |  |
| 35.Mastery of basic clinical skills (CPR, wound care) and interpreting ancillary test results (blood tests, imaging) |  |  |
| 36.Diagnosing and treating common diseases |  |  |
| 37.Knowledge of basic pharmacology for rational drug use |  |  |
