# Supplementary material for: Training needs and influencing factors among rural-oriented general practitioners in Chongqing, China: a cross-sectional survey and latent profile analysis
Source: Front Public Health. 2026 Jan 22;14:1743744. doi: 10.3389/fpubh.2026.1743744 (PMC12872738; doi:10.3389/fpubh.2026.1743744)
Supplement: Supplementary file 2 [file Table_2.DOCX]

| **Supplementary File 2**. Validation of the Psychometric Properties of the Chinese version of the Hennessy-Hicks Training Needs Analysis (Ch-HHTNA) Questionnaire | | | | | | |
| --- | --- | --- | --- | --- | --- | --- |
| Testing of content | | Specific indicators | Inspection standard | Ch-HHTNA questionnaire | | |
|  |  |  |  | scale A |  | scale B |
| **Item analysis** | | | | | | |
|  | Differentiation | Critical ratio (CR) | CR>3.0 and *p*< 0.05 | CR 10.26-27.22, *p*< 0.01 |  | CR 9.44-21.17, *p*< 0.01 |
|  | Homogeneity | Corrected item-total correlation (CITC) | CITC≥0.3 | all items CITC≥0.3 |  | all items CITC≥0.3 |
| **Validity analysis** | | | | | | |
|  | Content validity | Item-level content validity (I-CVI) | I-CVI≥0.78 | the I-CVI for clarity and relevance in the second expert consultation ranged from 0.857 to 1.000 | | |
|  |  | Scale-level content validity (S-CVI) | S-CVI≥0.90 | the S-CVI for clarity and relevance in the second expert consultation is 0.992 and 0.996 | | |
|  | Criterion validity (predictive validity) | Pearson’s correlation coefficient | coefficient value r_s_ , *p*< 0.05 | all research-related items (3, 6, 7, 9, 12, 14, 19, 23, 24, 26) in scale A were significantly positively correlated with "engaging in research work" (r_s_=0.109-0.183, *p*< 0.05), except for items 11 and 20. |  | only item 19 in scale B showed a significant positive correlation with "engaging in research work" (r_s_=0.137, *p*< 0.01), while other items were not statistically significant. |
|  | Construct validity (exploratory factor analysis, EFA) | KMO, Bartlett's test | KMO value≥0.6 and  Bartlett's test *p*<0.05 | KMO value = 0.957，Bartlett's test: χ^2^ = 16086.513, *p*< 0.001 |  | KMO value = 0.956，Bartlett's test: χ^2^ = 14082.298, *p*< 0.001 |
|  |  | Principal Components Analysis (PCA) | Effective extraction results were determined by: (1) eigenvalues >1, in conjunction with a scree plot; (2) each factor containing at least two items with loadings≥0.4; and (3) theoretical interpretability | 5 factors |  | 4 factors |
|  |  |  |  | two items (30 and 32) had cross-loadings but were assigned to corresponding factors after further analysis |  | multiple items having cross-loadings that lacked theoretical interpretability. |
|  |  |  |  | the results aligned better with the preset dimensions |  | the factor structure significantly deviated from preset dimensions and no theoretical interpretability |
|  |  | Cumulative variance explained ratio | ratio≥60% | 75.25% |  | 72.26% |
| **Reliability analysis** | | | | | | |
|  | Internal consistency reliability | Cronbach’s α coefficient | coefficient value≥0.7 | 0.979 in total, 0.841-0.958 for individual dimensions |  | 0.976 in total, 0.797-0.958 for individual dimensions |
|  | Split-half reliability | Spearman-Brown coefficient | coefficient value≥0.8 | 0.953 |  | 0.932 |
