# Supplementary material for: Training needs and influencing factors among rural-oriented general practitioners in Chongqing, China: a cross-sectional survey and latent profile analysis
Source: Front Public Health. 2026 Jan 22;14:1743744. doi: 10.3389/fpubh.2026.1743744 (PMC12872738; doi:10.3389/fpubh.2026.1743744)
Supplement: Supplementary file 4 [file Table_4.DOCX]

| **Supplementary File 4**. Variable coding in Logistic regression analysis | | |
| --- | --- | --- |
| Variable | | Assignment |
| **LPA group** | |  |
|  | Class 1 | 1 |
|  | Class 2 | 2 |
|  | Class 3 | 3 |
| **Professional title** | |  |
|  | Junior or less | 0 |
|  | Intermediate and Senior | 1 |
| **Parental status** | |  |
|  | No children | Z1 = 1, Z2 = 0 |
|  | One child | Z1 = 0, Z2 = 1 |
|  | Two or more children | Z1 = 0, Z2 = 0 |
| **Engaging in public health work** | |  |
|  | No | 0 |
|  | Yes | 1 |
| **Engaging in research work** | |  |
|  | No | 0 |
|  | Yes | 1 |
| **Monthly income level (CNY)** | |  |
|  | <3,000 | Z1 = 1, Z2 = 0 |
|  | 3,000~6,999 | Z1 = 0, Z2 = 1 |
|  | ≥7,000 | Z1 = 0, Z2 = 0 |
