# Supplementary material for: Training needs and influencing factors among rural-oriented general practitioners in Chongqing, China: a cross-sectional survey and latent profile analysis
Source: Front Public Health. 2026 Jan 22;14:1743744. doi: 10.3389/fpubh.2026.1743744 (PMC12872738; doi:10.3389/fpubh.2026.1743744)
Supplement: Supplementary file 5 [file Table_5.DOCX]

| **Supplementary File 5**. Training needs of rural-oriented general practitioners | | | | | | | | | | | |
| --- | --- | --- | --- | --- | --- | --- | --- | --- | --- | --- | --- |
| Skills | | Importance  (score A) | |  | Proficiency  (score B) | |  | Training needs scores (A–B) | | | |
|  |  | Mean | SD |  | Mean | SD |  | Mean difference  (95% CI) | t-value | Cohen's d | Dimension Mean (95% CI) |
| **Clinical Diagnosis and Basic Skills** | |  |  |  |  |  |  |  |  |  |  |
|  | 2.Utilizing information technology for electronic documentation and clinical data processing | 6.20 | 1.02 |  | 5.17 | 1.09 |  | 1.03 (0.92-1.14) | 18.24* | 0.81 | 1.26 (1.16-1.35) |
|  | 10.Providing specialized treatment and developing health management plans for patients | 6.07 | 1.03 |  | 4.86 | 1.09 |  | 1.21 (1.09-1.32) | 20.94* | 0.93 |  |
|  | 16.Assessing patients' psychological and social needs | 6.02 | 1.03 |  | 4.65 | 1.20 |  | 1.38 (1.25-1.50) | 21.58* | 0.96 |  |
|  | 18.Operating medical equipment (e.g., electronic sphygmomanometer, spirometer) using computerized office software | 6.22 | 1.03 |  | 5.16 | 1.17 |  | 1.06 (0.95-1.17) | 19.42* | 0.86 |  |
|  | 22.Assessing patient’s clinical needs | 6.04 | 1.11 |  | 4.79 | 1.12 |  | 1.25 (1.13-1.37) | 20.22* | 0.90 |  |
|  | 34.Managing common critical illnesses effectively | 6.31 | 1.01 |  | 4.78 | 1.20 |  | 1.54 (1.40-1.67) | 22.70* | 1.01 |  |
|  | 35.Mastery of basic clinical skills (CPR, wound care) and interpreting ancillary test results (blood tests, imaging) | 6.42 | 0.88 |  | 5.12 | 1.16 |  | 1.30 (1.20-1.41) | 24.36* | 1.08 |  |
|  | 36.Diagnosing and treating common diseases | 6.42 | 0.96 |  | 5.25 | 1.09 |  | 1.16 (1.05-1.28) | 20.33* | 0.90 |  |
|  | 37.Knowledge of basic pharmacology for rational drug use | 6.40 | 0.88 |  | 5.01 | 1.07 |  | 1.38 (1.28-1.49) | 25.92* | 1.15 |  |
| **Clinical Research and Evidence-Based Practice** | |  |  |  |  |  |  |  |  |  |  |
|  | 3.Critically evaluating published research | 5.13 | 1.33 |  | 3.78 | 1.35 |  | 1.35 (1.23-1.48) | 21.14* | 0.94 | 1.60 (1.49-1.72) |
|  | 6.Explaining personal research findings | 5.44 | 1.39 |  | 4.25 | 1.49 |  | 1.19 (1.04-1.34) | 15.94* | 0.71 |  |
|  | 7.Applying others or your own research findings to clinical practice | 5.64 | 1.29 |  | 4.25 | 1.44 |  | 1.39 (1.26-1.53) | 20.22* | 0.90 |  |
|  | 9.Identifying viable research topics based on clinical needs | 5.63 | 1.24 |  | 4.00 | 1.45 |  | 1.63 (1.49-1.77) | 22.51* | 1.00 |  |
|  | 11.Incorporating innovative ideas and cutting-edge knowledge into clinical practice | 5.86 | 1.09 |  | 4.37 | 1.24 |  | 1.49 (1.36-1.61) | 23.57* | 1.05 |  |
|  | 12.Conducting literature searches based on evidence-based medicine | 5.69 | 1.31 |  | 4.21 | 1.36 |  | 1.48 (1.33-1.64) | 19.29* | 0.86 |  |
|  | 14.Applying statistical methods to analyze personal research data | 5.56 | 1.35 |  | 3.78 | 1.58 |  | 1.78 (1.62-1.93) | 22.41* | 0.99 |  |
|  | 15.Clinical teaching, demonstration, and evaluation of colleagues/students | 5.69 | 1.23 |  | 4.09 | 1.45 |  | 1.59 (1.45-1.74) | 21.73* | 0.96 |  |
|  | 19.Writing personal research papers | 5.62 | 1.34 |  | 3.64 | 1.51 |  | 1.97 (1.82-2.13) | 24.36* | 1.08 |  |
|  | 20.Participating in health promotion-related research implementation | 5.65 | 1.23 |  | 4.12 | 1.47 |  | 1.52 (1.38-1.67) | 20.41* | 0.91 |  |
|  | 23.Collecting and organizing relevant research information | 5.78 | 1.23 |  | 4.10 | 1.37 |  | 1.69 (1.55-1.82) | 23.94* | 1.06 |  |
|  | 24.Designing research projects | 5.55 | 1.38 |  | 3.67 | 1.59 |  | 1.89 (1.72-2.06) | 21.84* | 0.97 |  |
|  | 26.Accessing research resources (e.g., time, funding, information, equipment) | 5.82 | 1.25 |  | 3.94 | 1.45 |  | 1.88 (1.72-2.04) | 22.59* | 1.00 |  |
| **Management and Organization** | |  |  |  |  |  |  |  |  |  |  |
|  | 4.Assessing the effectiveness of one's own work | 5.98 | 1.01 |  | 4.74 | 1.26 |  | 1.23 (1.12-1.34) | 21.60* | 0.96 | 1.56 (1.45-1.67) |
|  | 17.Managing time efficiently | 6.25 | 0.92 |  | 4.60 | 1.14 |  | 1.64 (1.52-1.76) | 27.00* | 1.20 |  |
|  | 21.Rationalizing the allocation of limited health resources | 5.98 | 1.06 |  | 4.38 | 1.30 |  | 1.59 (1.46-1.73) | 23.11* | 1.03 |  |
|  | 25.Collaborating effectively as a team with clear roles and responsibilities | 6.05 | 1.09 |  | 4.42 | 1.35 |  | 1.63 (1.49-1.76) | 23.63* | 1.05 |  |
|  | 27.Participating in administration and management tasks | 5.57 | 1.21 |  | 3.95 | 1.41 |  | 1.61 (1.48-1.75) | 22.74* | 1.01 |  |
|  | 28.Understanding healthcare system changes, familiar with policy norms, and skilled at process optimization | 5.78 | 1.26 |  | 4.13 | 1.39 |  | 1.64 (1.48-1.81) | 20.00* | 0.89 |  |
| **Public Health and Health Promotion** | |  |  |  |  |  |  |  |  |  |  |
|  | 29.Managing health records for key community populations (elderly, children, pregnant women, chronic disease patients) | 6.00 | 1.14 |  | 4.54 | 1.35 |  | 1.46 (1.33-1.59) | 21.89* | 0.97 | 1.47 (1.35-1.58) |
|  | 30.Providing health education to patients and the public using scientific knowledge | 5.97 | 1.18 |  | 4.64 | 1.28 |  | 1.33 (1.19-1.47) | 18.74* | 0.83 |  |
|  | 31.Assisting in public health emergency responses | 6.09 | 1.04 |  | 4.50 | 1.27 |  | 1.59 (1.46-1.72) | 24.35* | 1.08 |  |
|  | 32.Recognizing, reporting, and managing infectious diseases | 6.07 | 1.07 |  | 4.75 | 1.38 |  | 1.32 (1.18-1.46) | 18.72* | 0.83 |  |
|  | 33.Implementing immunization programs while managing adverse reactions | 6.07 | 1.04 |  | 4.43 | 1.26 |  | 1.63 (1.51-1.76) | 25.30* | 1.12 |  |
| **Communication and Interpersonal Relationships** | |  |  |  |  |  |  |  |  |  |  |
|  | 1.Establishing a good relationship with patients | 6.31 | 0.97 |  | 5.11 | 1.07 |  | 1.19 (1.09-1.29) | 23.44* | 1.04 | 0.90 (0.83-0.97) |
|  | 5.Collaborating harmoniously with colleagues and doctors in higher-level hospitals | 6.11 | 1.01 |  | 5.18 | 1.14 |  | 0.93 (0.83-1.03) | 17.83* | 0.79 |  |
|  | 8.Communicating with patients to promote joint decision-making | 5.97 | 1.12 |  | 5.19 | 1.16 |  | 0.77 (0.67-0.87) | 15.53* | 0.69 |  |
|  | 13.Giving effective feedback to colleagues and physicians at higher-level hospitals | 5.64 | 1.07 |  | 4.94 | 1.06 |  | 0.70 (0.61-0.80) | 14.21* | 0.63 |  |
| Abbreviations: SD, standard deviation; CI, confidence interval.  Note: **p* < 0.001. | | | | | | | | | | | |
