# Supplementary material for: Training needs and influencing factors among rural-oriented general practitioners in Chongqing, China: a cross-sectional survey and latent profile analysis
Source: Front Public Health. 2026 Jan 22;14:1743744. doi: 10.3389/fpubh.2026.1743744 (PMC12872738; doi:10.3389/fpubh.2026.1743744)
Supplement: Supplementary file 6 [file Table_6.DOCX]

| **Supplementary File 6**. Training needs difference univariate analysis of Latent Profile Analysis (n=508) | | | | | | | |
| --- | --- | --- | --- | --- | --- | --- | --- |
| Basic Information | | Total (n=508, %) | Class1 (n=236, %) | Class2 (n=209, %) | Class3 (n=63, %) | χ^2^/H | *p*-value |
| **Gender** | | | | | | χ^2^=1.977 | 0.372 |
|  | Male | 219(43.11) | 105(44.49) | 92(44.02) | 22(34.92) |  |  |
|  | Female | 289(56.89) | 131(55.51) | 117(55.98) | 41(65.08) |  |  |
| **Educational background** | | | | | | χ^2^=2.922 | 0.232 |
|  | Bachelor | 464(91.34) | 215(91.10) | 188(89.95) | 61(96.83) |  |  |
|  | Master | 44(8.66) | 21(8.90) | 21(10.05) | 2(3.17) |  |  |
| **Professional title** | | | | | | χ^2^=8.082 | 0.018** |
|  | Junior or less | 212(41.73) | 88(37.29) | 88(42.11) | 36(57.14) |  |  |
|  | Intermediate and Senior | 296(58.27) | 148(62.71) | 121(57.89) | 27(42.86) |  |  |
| **Career length (years)** | | | | | | H=4.739 | 0.094* |
|  | ≤ 3 | 178(35.04) | 80(33.90) | 68(32.54) | 30(47.62) |  |  |
|  | > 3 and ≤ 6 | 115(22.64) | 54(22.88) | 47(22.49) | 14(22.22) |  |  |
|  | > 6 and ≤ 9 | 164(32.28) | 73(30.93) | 78(37.32) | 13(20.63) |  |  |
|  | > 9 | 51(10.04) | 29(12.29) | 16(7.66) | 6(9.52) |  |  |
| **Marital status** | | | | | | χ^2^=3.231 | 0.199 |
|  | Unmarried | 270(53.15) | 120(50.85) | 110(52.63) | 40(63.49) |  |  |
|  | Married | 238(46.85) | 116(49.15) | 99(47.37) | 23(36.51) |  |  |
| **Parental status** | | | | | | H=6.464 | 0.039** |
|  | No children | 292(57.48) | 130(55.08) | 116(55.50) | 46(73.02) |  |  |
|  | One child | 172(33.86) | 88(37.29) | 71(33.97) | 13(20.63) |  |  |
|  | Two or more children | 44(8.66) | 18(7.63) | 22(10.53) | 4(6.35) |  |  |
| **Engaging in clinical work** | | | | | | χ^2^=1.797 | 0.407 |
|  | No | 70(13.78) | 32(13.56) | 26(12.44) | 12(19.05) |  |  |
|  | Yes | 438(86.22) | 204(86.44) | 183(87.56) | 51(80.95) |  |  |
| **Engaging in public health work** | | | | | | χ^2^=4.878 | 0.087* |
|  | No | 417(82.09) | 190(80.51) | 169(80.86) | 58(92.06) |  |  |
|  | Yes | 91(17.91) | 46(19.49) | 40(19.14) | 5(7.94) |  |  |
| **Engaging in teaching work** | | | | | | χ^2^=4.425 | 0.109 |
|  | No | 480(94.49) | 220(93.22) | 197(94.26) | 63(100.00) |  |  |
|  | Yes | 28(5.51) | 16(6.78) | 12(5.74) | 0(0.00) |  |  |
| **Engaging in administration work** | | | | | | χ^2^=2.41 | 0.300 |
|  | No | 451(88.78) | 215(91.10) | 181(86.60) | 55(87.30) |  |  |
|  | Yes | 57(11.22) | 21(8.90) | 28(13.40) | 8(12.70) |  |  |
| **Engaging in research work** | | | | | | χ^2^=7.448 | 0.024** |
|  | No | 488(96.06) | 232(98.31) | 195(93.30) | 61(96.83) |  |  |
|  | Yes | 20(3.94) | 4(1.69) | 14(6.70) | 2(3.17) |  |  |
| **Practice location** | | | | | | H=2.996 | 0.224 |
|  | The central urban area | 275(54.13) | 134(56.78) | 112(53.59) | 29(46.03) |  |  |
|  | The Wuling Mountain area | 75(14.76) | 32(13.56) | 35(16.75) | 8(12.70) |  |  |
|  | The Three Gorges Reservoir area | 158(31.10) | 70(29.66) | 62(29.67) | 26(41.27) |  |  |
| **Practice location^#^** | | | | | | χ^2^=2.356 | 0.308 |
|  | The urban area | 275(54.13) | 134(56.78) | 112(53.59) | 29(46.03) |  |  |
|  | The rural area | 233(45.87) | 102(43.22) | 97(46.41) | 34(53.97) |  |  |
| **Monthly income level (CNY)** | | | | | | H=8.697 | 0.013** |
|  | < 3,000 | 43(8.46) | 25(10.59) | 10(4.78) | 8(12.70) |  |  |
|  | 3,000 ~ 6,999 | 289(56.89) | 124(52.54) | 122(58.37) | 43(68.25) |  |  |
|  | ≥ 7,000 | 176(34.65) | 87(36.86) | 77(36.84) | 12(19.05) |  |  |
| Note: χ^2^ = Chi-square test, H = Kruskal-Wallis H test.*p < 0.10, **p < 0.05. ^#^The central urban area is categorized as 'urban', whereas the Wuling Mountain area and the Three Gorges Reservoir area are categorized as 'rural', in order to analyze the influence of urban-rural disparities on the training demand grouping. Class 1 = Low demand group, Class 2 = Medium demand group, Class 3 = High demand group. | | | | | | | |
